# Paediatric systemic inflammatory response syndrome (SIRS) and the development of patient-specific therapy: ethical perspectives through experts’ opinions

**DOI:** 10.3389/fpubh.2024.1420297

**Published:** 2024-10-30

**Authors:** Frederik Stolte, Silviya Aleksandrova-Yankulovska, Paul Thiemicke, Marcin Orzechowski, Catharina Schuetz, Florian Steger

**Affiliations:** ^1^Institute of the History, Philosophy and Ethics of Medicine, Ulm University, Ulm, Germany; ^2^Paediatric Immunology, Medical Faculty “Carl Gustav Carus”, Technic University Dresden, Dresden, Germany

**Keywords:** ethics-clinical, SIRS, personalised medicine, children, OMICS, artificial intelligence

## Abstract

**Background:**

Research for personalised therapies concerning the Systemic Inflammatory Response Syndrome (SIRS) in children involves the utilisation of OMICS technologies and Artificial Intelligence (AI).

**Methods:**

To identify specific ethical challenges through the perspective of healthcare professionals, we conducted 10 semi-structured interviews. The development of interview questions for the interviews was preceded by a systematic review of the scientific literature. To address the complexities of paediatric emergency research, informed consent, and data processing, experts with expertise in paediatric intensive care, computer science, and medical law were sought. After the transcription and anonymisation, the analysis followed established guidelines for qualitative content and thematic analysis.

**Results:**

Interviewees highlighted the intricacies of managing consent in personalised SIRS research due to the large amount and complexity of information necessary for autonomous decision-making. Thus, instruments aimed at enhancing the understanding of legal guardians and to empowering the child were appreciated and the need for specific guidelines and establishing standards was expressed. Medical risks were estimated to be low, but the challenges of securing anonymisation and data protection were expected. It was emphasised that risks and benefits cannot be anticipated at this stage. Social justice issues were identified because of possible biases within the research population. Our findings were analysed using current ethical and legal frameworks for research with a focus on the particularities of the patient group and the emergency background. In this particular context, experts advocated for an enabling approach pertaining to AI in combination with OMICS technologies.

**Conclusion:**

As with every new technological development, ethical and legal challenges cannot be foreseen for SIRS-personalised treatment. Given this circumstance, experts emphasised the importance of extending the ethics-legal discourse beyond mere restrictions. The organisation of supervision should be reconsidered and not limited only to the precautionary principle, which *per se* was seen as impeding both the medical progress and clinical flexibility. It was noted that the establishment and monitoring of guidelines were emergent and should evolve through an interdisciplinary discourse. Therefore, it was recommended to enhance the qualifications of physicians in the field of computer science, impart ethics training to AI developers, and involve experts with expertise in medical law and data protection.

## 1 Introduction

Systemic Inflammatory Response Syndrome (SIRS) presents a significant diagnostic and therapeutic challenge in the paediatric population (1, 2). Data on the prevalence of SIRS are inconsistent because it is rarely estimated alone but it is included in the complex group of sepsis (3). Current diagnostic criteria do not allow differentiation between infectious causes of SIRS and auto- or hyperinflammatory entities with diametrically different therapeutic regimens (4, 5). A lack of sensitive or specific diagnostic tools prevents early therapy. Additionally, scores such as the paediatric Sepsis-related Organ Failure Assessment (pSOFA) score and the Phoenix Sepsis Score were suggested (6, 7) but they cannot be used as screening tools for early diagnosis. Thus, the diagnostic process of SIRS depends mainly on the experience of clinicians. The need for clinical detection support systems has already been recognised in this context (8).

The analysis of the genome, epigenome, transcriptome, proteome, and metabolome, under the term “MultiOmics,” offers profound possibilities for the exploration of cell mechanisms (9), enhanced comprehension of the pathophysiology of SIRS, and better addressing of the clinical challenges (10, 11). Artificial intelligence (AI) and machine learning algorithms can further offer precise diagnostics and early subtyping of various infectious, autoinflammatory, and hyperinflammatory causes, thus forming the foundation for personalised disease management.

The application of MultiOmics and AI necessitates a nuanced consideration of the accompanying ethical aspects. Notably, there is a gap in the literature, as no studies have specifically addressed the ethical challenges of personalised research and disease management of SIRS in children (12). Considerations surrounding the research and treatment of SIRS focus primarily on the issues of patient autonomy and informed consent (13), whilst also delving into concerns related to beneficence and justice (14), aligning with the classical bioethical framework proposed by Beauchamp and Childress (15). Although parallels can be drawn between the autonomy and informed consent processes in SIRS and those in conditions like sepsis and other emergencies in children (16), it is still relevant to investigate whether there are unique ethical challenges presented for treatment decisions and participation in research projects involving SIRS (17). Medical decision-making and ways to empower children’s participation in research require special ethical scrutiny. In the case of SIRS, informed consent is usually obtained in an emergency context. Under life-threatening conditions, informed consent can be waived for adults (18, 19) and children (20). There are several suggestions regarding how to obtain informed consent for research in children. Should it be conducted in the classical form of parents’ consent and child assent, or should it be adapted to a dual consent procedure? (21). Furthermore, the involvement of minors in non-therapeutic research presents ethical controversy due to the concerns about reducing children to mere research subjects (17).

Further challenges involve the collection of genetic data in OMICS research. Following Regulation (EU) [23]/679 of the European Parliament and the Council, genetic data should be defined as personal data and, therefore, are subject to current data protection regulations (22). “Children merit specific protection with regard to their personal data,” (23) and further processing of previously collected and archived data is possible only under certain conditions (24). The purpose of personal data processing should be clearly defined in informed consent forms, as should the measures for data protection. The research subject should provide project-specific consent but other informed consent options are also discussed, such as consent-free data donation (25), broad informed consent (26), or re-consent for subsequent genetic research (27).

Ethical inquiries must also address AI design and application and its impact on individual well-being and public health. Algorithms powered by AI heavily rely on the quality and representativeness of the data upon which they are based (28). Arising ethical issues have been identified early, but not specifically for SIRS (29). In specific situations, AI can deepen health inequalities and heat non-discrimination debates. If AI were developed on restricted patient samples, which missed data for certain disease characteristics, it would perform poorly (30). Until now, positive research results have been released in regard to the application of machine-learning in paediatric care (31), but we noticed that the discussion surrounding the related ethical challenges offers more open questions than solid answers.

Thus, our study aims at the identification and analysis of specific ethical challenges concerning OMICS research with paediatric SIRS patients with specific consideration for AI-driven ethical issues in the strive for the development of personalised therapy for SIRS.

## 2 Materials and methods

In order to attain this aim, our objective was to assess the perceptions of healthcare professionals on ethical questions related to the ethical challenges of the project. We have conducted a series of problem-oriented, semi-structured interviews with 10 participants. The recruitment of interviewees involved consecutively searching and contacting potential participants with expertise in the areas connected to the research issue. Experts were selected according to their expertise in four areas: physicians with a specialisation in paediatrics and/or the treatment of SIRS; biomedical researchers with a specialisation in SIRS or similar diseases; informatics professionals with a specialisation in AI development or data processing; and professionals with a legal background and a specialisation in data security and/or AI-related legal research. Thus, eventually, seven experts were physicians, six of whom had direct intensive-care experience with expertise in precision medicine and OMICS research. One physician was specialised in biotechnology and molecular biology. Two experts had a legal background with a focus on medical data protection and the application of AI. One expert had a background in Medical Data Science with broad research expertise.

Semi-structured interviews are an established method of research in applied medical ethics. Through the application of this method, we were able to gain insight into the subjective perspective of our interview partners on the topic of the research. Moreover, semi-structured interviews provide flexibility in the conduct of the conversation, allowing for the possibility of *ad hoc* inquiries for clarification of interviewees’ statements or to initiate follow-up inquiries in order to focus on specific points mentioned during the interview (32, 33).

The development of themes and the specific questions for the interviews were preceded by a systematic review and ethical analysis of the scientific literature on the topic of the research. The results of this analysis have been presented elsewhere (12). Such a procedure allowed us to establish thematic areas that could raise ethical issues and dilemmas related to autonomy, the informed consent process, risks and benefits, AI-driven ethical issues, protection of privacy, social justice, and general suggestions for improvements.

The development of questions for the interviews was conducted by and intensively discussed by the interdisciplinary team of researchers. Interested interview partners were first contacted via email with an invitation to participate in the research. Throughout the recruitment process and at the beginning of the interviews, the interlocutors were informed about the project goals, the course of the interview, the voluntary nature of the participation, and the measures for the protection of their individual data. Only respondents who consented to the above requirements were interviewed. The interviews were conducted face-to-face, in German and English language, using a secure digital communication platform. Interviews were conducted by a researcher with a background in medicine and medical ethics.

Because the research objective does not include the influence of the personal characteristics of the interviewees on their opinions, no demographic data such as age, gender, or career path have been retrieved prior to or during the interviews.

Each interview was conducted based on the same questionnaire. Interviews were digitally recorded and transcribed. After transcription, the interviews were anonymized, i.e., all information that could reveal the identity of the respondents was deleted from the transcripts. The procedure for analysis followed established guidelines for qualitative content analysis (33, 34) and thematic analysis (35, 36). For the purpose of triangulation and in order to avoid bias in the results of the analysis, two researchers separately analysed the interviews and derived the main thematic categories touched upon by the interlocutors. The results then were compared. Thematic analysis was conducted according to the sequential phases prescribed for this method: (i) familiarisation with the data, (ii) generation of codes; (iii) search for recurring main and secondary topics; (iv) review of the themes; (v) definition of the themes; and (vi) reporting the results. After the initial analysis of the content, the saturation of the collected data was estimated as sufficient for the analysis. Therefore, no additional data collection was deemed necessary.

Throughout the interviews, recurring topics were specified and connected to representative quotes in order to illustrate the results. Based on the results of the analysis, a narrative synthesis was written.

## 3 Results

**Figure 1:**
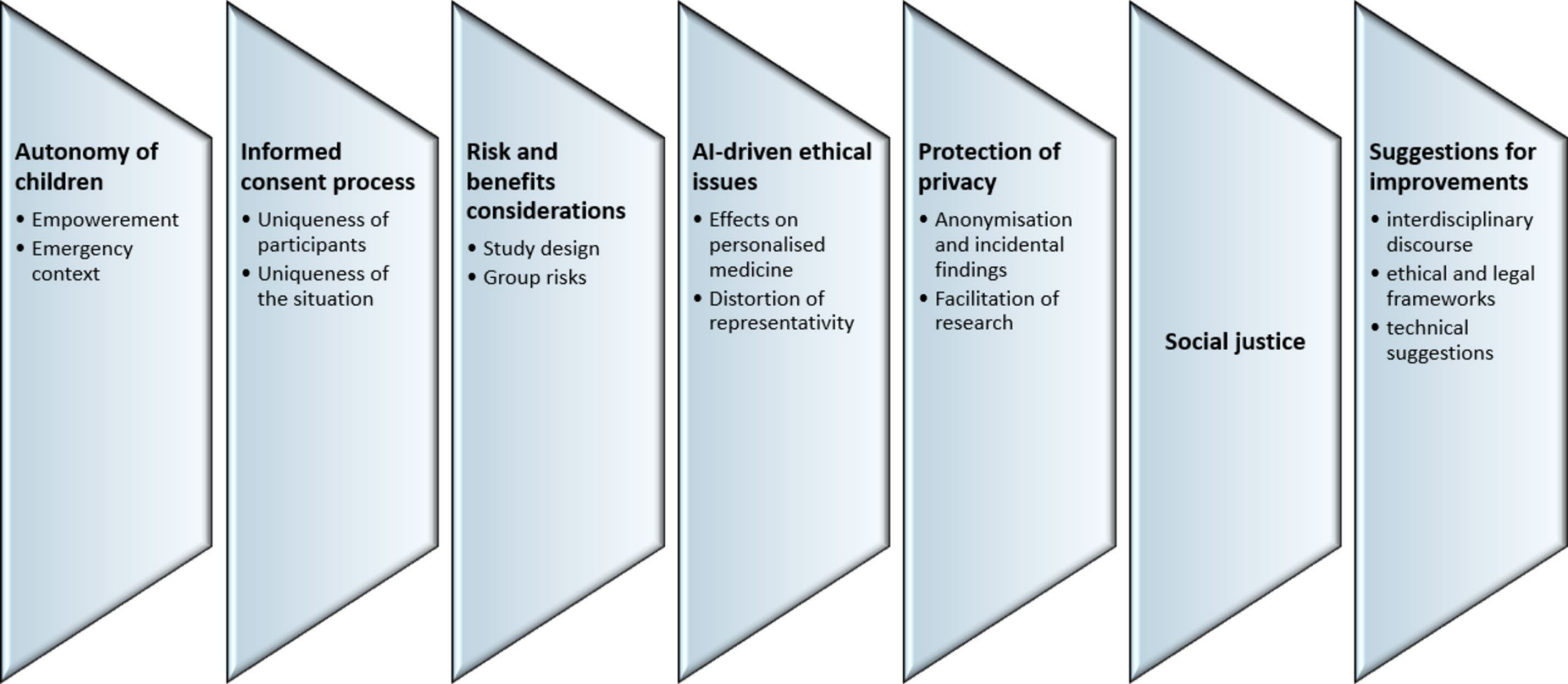
Major topics identified in the thematic analysis.

The analysis identified seven major topics, including (1) the autonomy of children; (2) the informed consent process; (3) risk and benefits considerations; (4) AI-driven ethical issues; (5) protection of privacy, (6) social justice; and (7) suggestions for improvements in research on personalised medicine. Within each topic, several subtopics can be identified (Figure 1).

### 3.1 Autonomy

All experts considered the empowerment of the children as important (*N* = 10). The informed consent process should prioritise engaging with the legal guardians, with due consideration for addressing the autonomy of the child. Thus, obtaining informed consent from children was seen as a general goal (*N* = 5):

*“I do believe that it is fundamentally important for patients to be able to participate in decision-making… an autonomous decision is possible for adolescents. However, this must of course be supported by the legal representative.” (i3).*

Furthermore, clinicians considered the practical implications of a child’s involvement in decision-making, as it also ensures their autonomy (*N* = 3), emphasising the active role of the child:

*“There should be a community, a community of those affected. Often, they [the children] cannot do anything, but it’s an active contribution against the disease. That’s why I would ask the child to give their consent for research data in a motivating way. And not just collect it, but say: ‘You are a part of this!’” (i8).*

Parental influence on children was viewed as inevitable in the course of upbringing; however, the experts did not consider it problematic (*N* = 2). Interestingly enough, two experts underlined that not only the autonomy of the child is under ethical consideration, but the autonomy of the legal guardians might also be questioned. When parents fear negative implications, such as inferior treatment, following eventual refusal to participate in research. In emergency situations where parents are inclined to consent to “everything” that promises at least some chance of improvement for their child.

In the context of emergency medicine, where SIRS belongs, experts thought that the autonomy of the child held little relevance. More important is the personal interaction between the physician and parents, and it cannot be encapsulated by guidelines. One expert went further, criticising ethics committees for their adherence to a limited concept of autonomy where only the autonomous patient is expected to give consent and must be informed before the procedure.

Three clinician-interviewees pleaded for a more pragmatic approach in accordance with the special context of emergency medicine:

*“Can a legal representative decide for someone who cannot get his future in his hands? If you say no, what is something easy to say, the consequence is that all research for critical care medicine and for infectious diseases is over.” (i5).*

Two experts believe that physicians may adopt a more paternalistic stance in situations where both the patient and parents are overwhelmed, acting not only as a professional but also as a compassionate human being. In such a case, the no harm principle and pure motivation should be led, i.e., no personal benefits for the physician should occur (*N* = 4):

*“I have learned significantly how the doctor explained to my 6-year-old son that he is truly sick and will be sick for the rest of his life… and I could never have told him that. The doctor did that, and we were just in the background.” (i8).*

Generally, the proxy decision-making for the child was deemed problematic (*N* = 2). However, a situation where the will of the child and the decision of the legal guardians differed did not occur to any interviewee, indicating its little practical relevance:

*“I myself have not encountered such a situation, but I would not accept the parents’ consent alone, if the child does not want to participate.” (i10).*

If children understand the aim of the research, experts involved in the project see it as important to accept their decision, whether they choose to participate or refuse, regardless of the legislation.

### 3.2 Informed consent process

All interviewees agreed that **the uniqueness of the participants** must be addressed (*N* = 10). The dynamic interaction between the physician, child, and legal guardian presents three realms of understanding. The responsibility for shaping these interactions should lie within the individual decision-making of the medical specialist. All experts pointed out that children’s perspectives do not equate to those of adults. Some interviewees drew parallels to their own children:

*“I have a four-year-old at home, so if I ask my four-year-old something that is my work-related, she will answer something that is from her world. Those questions and answers are not aligned.” (i1).*

The importance of the physicians’ empathy and the active role of the child was emphasised over the focus on the information delivered. On the other side, though, one legal expert considered age-appropriate handling as a requirement, as it is stipulated in the German Civil Code (Bürgerliches Gesetzbuch § 630e) that the information must be understandable (37). Instruments designed to inform children that provide sufficient adaptability without impeding the physician’s assessment were greatly appreciated (*N* = 10). Pictures and other tools should be provided, consistent with the maturity of the child.

*“We know from communication science that images are much more readily perceived by the brain than text. Therefore, I see these visual consent and information mechanisms as a great opportunity. Especially if it is presented in a child-friendly manner, I would say.” (i9).*

One expert viewed video clips, picture books, and images that were especially useful for younger children. Accordingly, two physicians have set the age limit practically to 8. For individuals under 8, the emphasis was using pictures in the process, whereas for those above 8, additional information should be provided. Furthermore, two interviewees suggested that instruments should also consider the gender of the child, especially during puberty. They believed that the information materials may include graphics and statistics in a child-friendly manner to explain the use of AI and research goals. The interviewees had little experience with tools for assessing the maturity of children, though they saw a potential benefit of such in non-emergency situations. Currently, such instruments play no role in their experience with informing children. The practical insignificance of the assessing instruments was further stipulated in the opinion of one legal expert, who pointed out that those scores hold no significance for the legislator.

Secondly, the uniqueness of the situation is important for the informed consent process. Experts agreed that only the physician’s assessment can address emergency situations, unique social circumstances, or the understanding of legal guardians properly (*N* = 9). Interviewees with a legal background (*N* = 2) pointed out that current legislation is therefore short on guidance on how to regulate such specific situations.

“*I would take into account the gravity of the situation, so if it’s dramatic, express that clearly [in the conversation]. Especially with children, it should not be communicated in “baby talk.” […] From my subjective experience, priority should be to understand the child’s distress.” (i8).*

In emergency situations, it was seen as imperative to provide only the most essential information (*N* = 10):

*“Only the most crucial information, because it’s a situation where the child most likely is not in full senses, let us say, and then the parents are terrified in most of the cases.”(i1).*

Experts emphasised that extended content does not lead to better-informed patients. The lack of direct benefits, if that is the case, should be explicitly communicated to participants in research and they should further be assured of the best standards of treatment, even if they refuse participation. One expert saw it as critical to inform the public that no harm would be done through participation and to clarify the extent of the data examination. As a general timeframe for informing, five to ten minutes were seen as appropriate in an acute emergency situation (*N* = 1). Interviewees from the professional area of intensive care encountered challenges in timely information delivery due to emergency situations. They deemed it appropriate to notify legal guardians within the first 24 h, including details about participation in the research project (*N* = 5). Back to the measurement of maturity, interviewees thought that it had no significance in the emergency context (*N* = 5). Similarly, tools such as pictures are negligible in emergency situations (*N* = 4). Legal experts (*N* = 2) recommended that images or other tools be used after the acute situation has been handled. Other experts (*N* = 4) pointed out that information should be delivered to the child after the emergency has been addressed.

Informed consent for research in an emergency context presented specific challenges. The intensivists considered it justified to collect blood for study purposes in emergency situations, provided that the parents are informed of the next possibility. Physicians emphasised that emergency therapy and emergency diagnostics cannot be artificially separated from study participation (*N* = 4):

*“Well, I would say we have a relatively short timeline where we need to act. […] The question always is, what is the burden on the patients? […] Or we have to draw blood anyway. But that’s still within limits, where ultimately the patient is not excessively burdened, right?” (i2).*

Contrary to popular belief, legal experts saw a necessity to, at least formally, separate emergency medical indications from research (*N* = 2). If non-essential blood samples for research purposes need to be taken and the legal guardian cannot be informed for various reasons, the decision should be weighed on the purpose of the research question and the expected risks concerned. One physician argued accordingly, saying that these questions should be addressed in advance of each project.

### 3.3 Risks and benefits

As the first sub-topic, the challenges within the study design were pointed out. The drawing of additional blood was considered a negligible risk by the interviewed physicians and researchers (*N* = 8):

*“If we are drawing one five-milliliter tube or two five-milliliter tubes, I do not think that this actually makes a difference to the child or to the parents.” (i1).*

The small cohort size was acknowledged as problematic for the results by six experts, but one pointed out that there might be instruments of data science to mitigate that limitation.

*“I think that the major problem is that our “n” number is still very, very small, and there are gazillions of covariables, as we are thinking about. Every single one of us is a biological individual. My physical state today is very different from what it will be tomorrow, and all of those covariables will be there.” (i1).*

Two interviewees felt that this was less problematic, as participation may be sufficient for a precision medicine approach. All participants (*N* = 10) agreed that further studies would be needed. The mitigation of subjective distortions and opinions can be accomplished through the implementation of rigorous inclusion criteria and well-executed protocols (*N* = 3).

Furthermore, the definition of SIRS was considered problematic, as it remains in use in paediatrics but was abandoned for adults in 2016 (*N* = 1). Therefore, the age-related cut-off for participants was perceived as purely arbitrary, pointing out that concepts are defined strictly, whilst biological and physiological differences may change gradually.

Group benefits were apparently recognised through identifying aetiologies through a MultiOMICS approach with the ultimate goal of understanding SIRS comprehensively and minimising inappropriate therapies (*N* = 8). In this sense, the group benefits were estimated as high on the basis of the negligible risks of additional blood sampling. Specifically, one expert anticipated the OMICS studies to contribute to obtaining more insights into the state of immunoparalysis in children. In the future, the utilisation of a patient’s genetic identity, genomic score, or combination may become the standard for enrolling patients.

*“So, I believe that a project like yours would contribute or be predestined to demonstrate to politics what is actually possible with research.” (i9).*

However, it was recommended (*N* = 3) that in the aftermath of the project, it should be assessed whether the personnel, time, and financial investments are justified in consideration of the therapeutic benefits.

### 3.4 AI-driven ethical issues

Risks deserving special attention in the context of personalised therapy development are AI-induced risks. Thus, the significance of these risks justified the formulation of a separate AI-related topic.

Regarding AI-effects on personalised medicine, AI can lead to a departure from the individual approach in medicine, as the physician may be induced to only act based on algorithms that are more or less representative (*N* = 2). Personalised therapies always involve the possibility of obtaining information about the entire genetic fingerprint and identity of the patient. Interviewees pointed out that questions that were previously relevant only in research will now play a role in personalised therapy counselling and impede self-determination (*N* = 2). This requires the extension of the stakeholders so that we respect the opinion not only of the triad physician-legal guardian-patient but also of governments and various patient and legal organisations. One interview partner recognised the risks associated with defining too small stakeholder groups.

The AI-driven distortion of representativity was separately addressed in direct association with issues of justice. Interviewees were aware that AI can lead to changes in the assessment of reality, as it is dependent on data quality (*N* = 4). Thus, a lack or insufficiency of data could lead to biases:

*“To set up an AI, that’s not enough. It is said that training models currently require 200 to 1,000 datasets to train a model. And that’s just the training model.”(i8).*

Physicians saw AI-aided decision-making as useful for special therapeutic situations, although it may not always capture the subtleties of highly individual situations (*N* = 2). They assessed it as a tool generating objectivity when used within its limitations, and the physician, with his accumulated experience and knowledge of the situation, still manages the treatment. AI was also seen as a tool that can help mitigate the impact of covariables that may alter research results, and as such, AI was perceived to be an instrument of justice (*N* = 3). These interviewees emphasised that AI should remain transparent and controllable. The underlying logic and algorithms should be comprehensible for users and for those affected.

### 3.5 Protection of privacy

The challenge, of how to avoid traceability of individual data was discussed. All experts agreed that complete anonymisation should be ensured (*N* = 10). However, regarding OMICS research, there was disagreement, how this could be achieved, especially regarding incidental findings.

*“I would simply try to ensure that no conclusions can be drawn about the individual… which, of course, can still be achieved through genotyping in the case of abuse… That would be possible. The important thing is not to go back to the child, even if it has a mutation that you can treat. Instead, you should appeal: “If you have this, just get in touch again.”(i8).*

Another medical expert also expressed the opinion that backtracking should be possible if a direct treatment is available. Research participants should be informed of the results upon the end of the project, even if personal data, such as telephone numbers and addresses, need to be used:

*“We also try to find a solution for this, because if we find some pathogenic mutations for another disease and not for an underlying disease, and …. we will explain the status. We will discuss with the patient and also legal guardians the effect of these pathogenic mutations, if it’s necessary to consult other subspecialists, then we will do this.”(i7).*

In direct contradiction, one interview partner considered the implementation of anonymisation of genetic data as impossible, if therapeutic interaction was envisaged:

*“I believe there are too many possibilities for abuse, and it requires too much regulatory and technical effort to handle it. Therefore, I would simply make a brutal cut and hope for the best.” (i8).*

Other interviewees stated the procedure should strictly refer to informed consent (*N* = 4). Incidental findings should be communicated if the legal representatives wish it, as long as the child is not of legal age (*N* = 2). One expert emphasised that incidental findings should not be communicated unless there is a therapeutic consequence. If they are to be discovered, it should be optional for study participants to be informed. Therapeutic consequences should be evaluated based on the presumed improvement in the patient’s quality of life, which depends on the physician’s familiarity with the patient, contradicting anonymisation. One interview partner suggested a practical approach to inform the legal guardians automatically, because it is in their free will, whether they open the letter, or not. Informed consent should therefore be project-specific (*N* = 4). Two physicians advised that diagnostics should, if possible, align with the research hypothesis and be investigated only in that regard. Most experts emphasised that Data ownership should stay both with the legal guardian and the child, with the latter being sensitised and informed about the storage and processing (*N* = 8). One of the interview partners believed that in this context minors should only receive the data when reaching the age of 18, though this might be not of therapeutic benefits to the child.

Protection of data from misuse by insurance companies and corporations should be ensured (*N* = 10). Data holds value for individuals and communities, including companies, and managing these interests requires careful navigation. There should be widespread public awareness about data protection (*N* = 9). Access should be restricted to establish a secure environment for data processing (*N* = 7). Such restrictions may concern the location, where the data can be analysed (*N* = 3), or may include special institutions for data processing (*N* = 2).

Further, the issue of how research could be facilitated whilst respecting individual rights of data protection was raised. Most interviewees agreed that the re-utilisation of data for future purposes should require a renewed informed consent procedure (*N* = 6). One legal expert found this unnecessary, as long as there is a permanent option to revoke consent and one physician emphasised that follow-up studies should be possible without re-informing the participants. The interviewees with a legal background generally found this question to be more difficult to answer (*N* = 2). Anonymous data, where tracing back to the individual is impossible, should be allowed for use in other research projects as long as they have a public or community benefit. But this raises the question of whether genetic data is not always retraceable to some extent. If this is the case, a renewed informed consent procedure is envisaged. In the case of study design with broad consent for data use, the legal experts believed that there was no need for further information or consent from the participants, as long as complete anonymisation is ensured. Most experts (*N* = 6) agreed that aggregated anonymised data may be usable for additional projects depending on the purpose of the research without the need for a renewed informed consent procedure, provided that ethical principles are adhered to. In their opinion, in specific cases, the data should be accessible to everyone (*N* = 2):

*“In six years from now, others repeat a similar approach. They find something which they could not expect and then they may retrieve our data from the web. They realise that actually they have found exactly the same thing, but only we were not aware of how to analyse the data.” (i5).*

Legal experts considered the possibility of changing the purpose of a research project without renewed consent to be highly problematic. Safeguarding against misuse, including the use of data for other research purposes in subsequent studies, was therefore emphasised (*N* = 2).

*“The data protection regulation requires an upfront definition of the purpose, and if I want to change the purpose in the end, I must meet stringent requirements.”(i9).*

### 3.6 Social justice

Social justice was seen as equal chances of inclusion of different population groups in research. Seven interview partners saw socioeconomic differences as a challenge to ensuring justice across various populations and communities. It might impede inclusion but also influence nutrition, gut microbiota, and lifestyle that merge into a condition that potentially alters results unfavourably. Focussing on these social justice issues was deemed challenging due to their inseparability from other contributing factors:

*“There are just so many* var*iables. Most likely, this is something that we will see from the data. Then we need to be academic professionals and then be able to say, “Hey, I think that this is what we see, and then this is what we need to consider.” (i1).*

Two interviewees saw discrimination as necessary to the approach of precision medicine, in which personalised treatment is the focus. As inherent in the study design, discrimination among different subgroups should not be simply seen as an issue, but as a result of a precision medicinal approach. One interviewee judged this issue, therefore, as negligible, as smaller, more specific cohorts will be identified anyway and further investigated over time. Generally, interview partners agreed that confounding biases should be evaluated afterwards and further studies needed to be conducted (*N* = 7). Two experts viewed the exclusion of specific patient groups as an unsolvable problem that only advocates for the inclusion of as many patients as possible, as long as they meet the criteria for participation. The potential exclusion of certain groups from a study should therefore be weighed in favour of the possibility of new therapies and the avoidance of futile treatments (*N* = 4). Another interview partner argued that since there is no legal entitlement to the best treatment and various economic interests exist, no changes in the study designs should be made to prevent the exclusion of potential socioeconomic groups.

The limitations of personalised therapy were further related to social justice issues. In the opinion of six experts, individual and ethnocultural factors may influence outcomes in personalised treatment, especially considering the small sample size and the diverse populations. One medical expert, on the contrary, perceived that there were no significant challenges associated with the heterogeneity of patients.

Three physicians considered in this context that the term “Personalised Medicine” needs to be explained to the patient since, at the moment, truly personalised treatment is impossible; it rather involves the transfer of research results, i.e., genetic patterns, from small study cohorts to the genetic individual.

### 3.7 Suggestions for improvements in research on personalised medicine

All experts agreed that an interdisciplinary discourse should be promoted, addressing weaknesses and biases in research towards the development of personalised therapy for SIRS (*N* = 10):

*“In Europe, it is quite challenging. We need to come together and ask ourselves: What kind of research do we want to enable? In consensus.” (i9).*

Two interview partners emphasised that the final judgement on the justice and risks of decision-making based on genetic or genomic characteristics must be reached by governments and through interdisciplinary consensus. Another expert went further, pointing out that political decision-makers should define the general purposes for which OMICS research should be allowed. Other professional groups with legal experience could be consulted when it comes to information about data processing and legal claims (*N* = 3). More specifically, the monitoring of compliance with the data protection regulations of the European Union (EU) should be ensured by specific data protection managers and data protection officers (*N* = 2). An interviewee with a background in computer science emphasised the role of physicians in that regard:

*“Medical professionals should not be afraid to examine the data and assess the IT infrastructure and how it is being handled.” (i8).*

Therefore, ethical and legal frameworks should be customised (*N* = 6) in several aspects: (a) specific guidelines in the informed consent process for children at various ages (*N* = 4); (b) ethical considerations governing the use of AI and big data should be project-specific (*N* = 2); (c) ethical considerations should differentiate between the research, the registration process for a drug/immunotherapy, and the implications for daily clinical practice (*N* = 2).

Ethical and legal considerations should not be artificially separated from each other (*N* = 2):

*“These are specific guidelines that need to be established, and you are correct that they include ethical provisions. However, this can quickly become a legal requirement, namely when I stipulate this [in legal sense]. Therefore, I see ethics and law closely intertwined here.” (i9).*

Various legislations, procedures, and rules between different countries in multinational projects present numerous complications (*N* = 3). Therefore, a uniform approach to data processing would significantly facilitate research. Three medical professionals took a stand against any further tightening of legal restrictions, as they considered them sufficient.

The heterogeneity of the small patient cohorts could be addressed by changes to General Data Protection Regulation (GDPR), allowing larger segments of the population to participate in research projects in general (*N* = 1). In addition, the anonymisation of OMICS samples was considered problematic (*N* = 3).

Experts emphasised that the combination of OMICS research, AI application, and precision medicine is a novel concept, and that, guidelines should be extremely broad-minded in their initial stages. They should adapt and evolve alongside medicine, which is a practical science (*N* = 9).

*“Actually, we are facing something which is fully novel. There are no gold standards.” (i5).*

Furthermore, several technical solutions were proposed. Digital twins, i.e., digital models of an actual individual, could be established for younger children to provide age-appropriate information (*N* = 1). As a solution for further self-determination in the use of data, an app was suggested by one interview partner that keeps individual patients informed and allows for potential renewed consent. Anonymisation and further information regarding the purpose of the research would therefore not be mutually exclusive. Every participant in a study could be informed through an app without the researchers being notified.

*“So, if I have my data and I allow it to be used in* var*ious research contexts, then I could, for example, imagine that there is an app that sends me a push notification and informs me in what context these data can/could/should be used.” (i9).*

A right to object could also be implemented in this app. As a solution to representativity, Electronic Health Records and institutions in the form of data altruistic individuals, which could receive data donations, could be implemented (*N* = 1). Challenges related to the use of AI in research, such as lack of representation or issues of injustice, may be addressed by the application of synthetic data to be a solution to better train discriminatory AI and address issues of justice:

*“And the other thing, of course – I am a big fan of synthetic data – is to create a larger cohort from a small cohort to develop AI models […]. This can also be attempted synthetically. Furthermore, a strong medical insight should accompany the data analysis.” (i8).*

## 4 Discussion

Our study aims at the identification and analysis of specific ethical challenges concerning personalised research with paediatric SIRS patients. The intricate combination of underlying diseases, which often results in paediatric emergencies, coupled with clinical research that lacks direct participant benefits and incorporates OMICS and AI technologies, necessitates our scientific aim.

The first and second identified topics concern the autonomy of research subjects and the particularities of the informed consent process *per se*. The prioritising of the legal guardians in the informed consent process without impeding the autonomy of the child was considered important by our interview partners. However, this cannot be artificially separated from family dynamics, as shown in other studies (38). Possible influences of legal guardians on their children were not deemed problematic by interviewees, as it should be a joint decision, if possible. Informed consent was preferred over assent, but it was not always implementable in real-world scenarios. Since the terms informed consent and assent have legal, ethical, and philosophical dimensions, we focus on the definitions as they are discussed in the European Clinical Trials Regulations (39). Consent is associated with autonomy, full legal competence, and the capacity to willingly participate after being informed about all relevant aspects of this decision. This ability is granted at varying ages from 15 to 18 by the different countries of the EU (40). Assent incorporates the lack of that ability for minors. Nevertheless, it is understood as a legal requirement, corresponding to a dual-consent procedure, but alone is not sufficient for participation. The requirement for assent for participation varies between member states of the EU, as it is age-dependent. The autonomy of children was valued higher than could be expected in an emergency situation. Three interviewees would not accept the decision of legal guardians, if children, who were deemed to understand the risks and aims of the study, declined, regardless of legislation or age.

The legal guardians’ right to surrogate decision-making is limited in the case of a child’s participation in research. In this particular situation, if the child refuses to be involved, this should be taken into account, in the same line of human rights protection as is the content of Article 6 of the Oviedo Convention (41). The prevailing presumption here is that participation in research bears known and unknown risks versus unguaranteed benefits. On top of this, the subject of research is the child and not the legal guardian, as the law intends to protect the child’s wellbeing. Since the Oviedo Convention was not ratified by all European countries, including Germany, general principles concerning human rights were used. For example, German ethics committees refer to Emanuel et al., but they adopt this perspective in the context of fair patient selection with the right to decline due to special vulnerability (42). Similar principles are postulated in the guidelines of the EU concerning clinical trials involving minors, which emphasise respecting the child’s right to decline (39).

However, in emergency situations, the legal guardians must engage in surrogate decision-making on behalf of their child, although their autonomy and medical literacy may be limited. Therefore, experts suggested that physicians should take a more paternalistic approach, emphasising not only their professionalism but also compassion and ethical competencies, which refer to the fundamentals of every physician-patient relationship (43). The proxy decision making for the child was deemed challenging by our interviewees in a research context without direct benefits for the participants, but should be addressed practically. This perspective is confirmed by studies, investigating the opinions of parents regarding research that lacks direct benefits (17). Hypotheses of reducing children to mere study objects, if no informed consent was possible, were therefore criticised as unrealistic and detrimental, as special necessities of emergency research are ignored. It was emphasised that the realities of emergency research should not be misjudged with arbitrariness (44).

All experts emphasized that the uniqueness of participants and situations must be addressed in the informed consent process. The empathy for the distress of child and parent and the conveyance of security was specially considered by interview partners, who drew parallels to their own children. Sufficient adaptability was seen as essential and tools to deliver information should be child-friendly and understandable. It aligns with general ethical principles of patient rights (39, 41) and an improvement through new instruments was also proposed in recent studies (45). Understanding the world of the child and the legal guardian as a layman was therefore identified as a challenge in the context of research towards the development of personalized therapy for SIRS. The perception that the increased quantity of information would lead to better-informed participants, was qualified as a misconception, whereas new technologies such as OMICS and AI necessitate larger volumes of content. Experts were focussed on enhancing the understanding of the information. In this respect, aids such as picture books, images, videos, and digital instruments were greatly supported. They were considered more negligible during emergency situations, but were rated useful afterwards. These standpoints fully align with current trends of deeper and more extended involvement of children in the informed consent process and the development of triadic (physician-parents-child), instead of dyadic (physician-parents) decision-making for children-patients, particularly with the help of visual aids supporting the process of informed consent (46, 47).

In contrast, the assessment of the maturity of the child by empirical scores was unanimously rejected by our experts on the grounds that they offer little flexibility in emergency situations and thus for most of the SIRS patients. Narrow constrictions through guidelines were seen as especially perilous, in line with other analyses (48). Furthermore, scores were seen as meaningless, as they were not addressed by legislation or its strict age limits. Interview partners with a legal background emphasised that legislation will not be able to manage the highly specific conditions of SIRS, as demonstrated by the variety of age limitations for decision-making capacity established in Europe (40).

Some experts focussed on the time frame of informed consent, whilst other interview partners focussed on the content, i.e., the absence of direct benefits, the assurance of uniform treatment, and that no harm was intended. Legal experts added as essential information on how far the examination of the data goes. Interview partners with a background in Data Science emphasised information about AI and statistical methods as crucial. All of these aspects are covered broadly in the international legal and ethical guidelines (19, 20, 49) but they pose specific practical challenges. Guidelines seem to fall short for these particular issues despite stating them broadly. There is a recognized demand for an augmented flow of information regarding OMICS and AI technologies to facilitate its understanding. Despite the constraints of time, it becomes evident that there is a need to establish guidelines encompassing informed consent for personalized SIRS research.

Withholding consent was seen as applicable and generally approved by the interviewed physicians in cases where timely information was impossible. Still, a dual-consent procedure for informing the child and legal guardians during and after the emergency was seen as desirable. In recent studies, this procedure has received the approval of patients and intensive care specialists (13, 50). As physicians saw negligible risks of participation through drawing blood, they therefore tended not to separate emergency diagnostics and therapy from research participation. This is supported by Katz et al., who identified the same ethical foundations for critical care and research (49). In our interviews, legal experts considered it more important to, at least formally, make distinctions. Informed consent regarding research should be received in advance, though not all samples will be taken. If this may not be feasible, the estimated purposes of the research and the expected risks should be evaluated in advance, and the participants should be informed as soon as possible. This estimation of various risks and benefits aligns with the ethical principles of the Declaration of Helsinki addressed in studies, searching for challenges of enrolling nonautonomous intensive care patients in clinical studies (51). Also, we must distinguish between therapeutic, experimental, and non-therapeutic research. Whilst for non-therapeutic research all of the above stands, that is not the case for the application of experimental therapy for research purposes. Referring to the cornerstone ethics document regulating research with humans, the World Medical Association (WMA) Declaration of Helsinki, a physician can use unproven therapeutic intervention, so to say to perform a single experiment, only with the consent of the patient or the patient’s legal representative and if “in the physician’s judgement it offers hope of saving a life, re-establishing health or alleviating suffering” (52). The text of Article 8 of the Oviedo Convention is in the same spirit, saying that: “When because of an emergency situation the appropriate consent cannot be obtained, any medically necessary intervention may be carried out immediately for the benefit of the health of the individual concerned.” (41). Thus, emergency therapeutic research is only possible under certain conditions amongst which first and foremost are informed consent and the direct benefit, i.e., experimental therapy as a life-saving means of last resort (53). Such a decision usually requires the involvement of a group of physicians. With the development of the personalized therapy for paediatric patients with SIRS, we are still at the stage of non-therapeutic research, thus, a regulatory framework for therapeutic experiments is not relevant yet. However, we must still keep in mind the highest level of precaution within the EU legislation in this regard. The directive [54]/20/EC of the European Parliament and the Council on clinical trials for medicinal products does not include an exception for emergency circumstances but requires informed consent in all cases (54).

Challenges for research with children without direct benefit in emergency situations were addressed by our interviewees and risks of blood sampling were deemed negligible. The risk–benefit ratio appeared to be very favorable in view of the aim to identify the root causes of SIRS through the MultiOMICS approach and eventually to gain a comprehensive understanding of this disease. In a further perspective, such research is expected to minimize the stress induced by false, non-personalized, therapy and to reduce SIRS mortality rates. Interviewees considered the benefit of our particular project to be a trial for further research. As the utilization of a patient’s genetic identity was expected to become a standard in the future, concerns were expressed about resource allocation for the provision of innovative treatments.

The danger of a distortion of reality precludes the application of AI models because they are dependent on the disposability and quality of data. To address this challenge, ethical guidelines from the EU have already pointed out three dimensions of trustworthy AI (55). It should comply with current law, adhere to ethical principles related to autonomy, and prevent harm. Fair AI is regarded as an equitable distribution of benefits and costs. It should exhibit robustness in terms of technical reliability, and social representation must be ensured, encompassing vulnerable groups such as children. Transparency, the opportunity to explain and understand key mechanisms, and a self-determined handling of data and privacy protection are therefore key elements. An institutional monitoring of impacts on society and clear assignment of responsibilities were recommended. Furthermore, a self-assessment test designed as an operational tool, which is intended for use by a multidisciplinary team, was published in 2020 (56). This includes a questionnaire that addresses the abovementioned aspects. A regulatory framework is currently in development to ensure that AI meets these requirements referred to as “EU AI Act” (57). In summary, we could not help noticing that these publications focus on the precautionary principle, as medical research, especially OMICS research, remains understudied. Most studies cover algorithmic challenges cementing inequalities, but few deal with the wider empirical impact of AI on patients and the healthcare system (58). However, during the course of conducting qualitative interviews, it was observed that the positive implications of AI were significantly more prominent than initially anticipated (30). This may indicate that ethical considerations tend to overanalyze risks and overlook chances since empirical consequences cannot be foreseen yet. That holds especially true when addressing these issues through technical approaches (58, 59). The utilization of synthetic data was recommended, with interviewees proposing an interdisciplinary approach, additional training of AI models, and a nuanced interpretation of their alignment with reality. Literature addresses these technical possibilities, such as the increase in diversity of data through globally trained AI models. The monitoring of medical algorithms through special algorithm-interpretability techniques was also discussed in recent studies. These considerations met the general critique of experts interviewed in this study. They underlined that current frameworks and legislation are limited to restrictions and should also include enabling factors, especially for the use of AI. Their ethical considerations were therefore aligned with the principles of Vayena and Blasimme’s “systemic oversight” (28), who see neither the precautionary principle nor a “wait and see” approach as ethically justifiable (60). Their more emergent approach meets the opinions of the interviewed experts, regards similar principles formulated in the ethical guidelines of the EU (55), and is in general enough to be tailored to the unique aspects of SIRS. Furthermore, its six cornerstones, adaptivity, flexibility, inclusiveness, responsiveness, reflexivity, and monitoring, i.e., the acronym “AFIRRM,” are developed for biomedical research and also refer to OMICS diagnostics (60). Ethics committees should be opened for new professions and govern, evaluate, and evolve actively and in parallel with the progression of technology. Therefore, the medical perspective provided by clinicians is considered equally important as that of technology developers. We noticed that physicians frequently expressed a lack of expertise concerning AI and Big Data in the interviews, often suggesting consulting directly with professionals in these disciplines, whereas an expert with a background in computer science emphasised that physicians should take a more active role in the shaping of AI. To address this fairness issue of AI, the active role of medical professionals was also seen as essential in recent studies, emphasising the view of our interview partners (58, 59).

In all our interviews, the tension between informational self-determination and research freedom and progress was an issue. Complete anonymisation should be applied, but it was seen as impossible if therapeutic interventions were envisaged. Especially the handling of incidental findings was seen as controversial. Literature shows that the intricate interpretations of incidental findings hold their own fallacies (61) and counteract anonymisation. The extensive ethical discussion concerning the management of incidental findings focuses on general ethical principles and is not specific to SIRS and OMICS research (62, 63). However, it should consider these factors. An analysis of all biological processes that define an individual presents a significantly more precise method for re-identification as compared to conventional genetic analysis. German ethical councils address this challenge with the call for additional structural requirements, technical inventions to secure anonymisation, and further participation in governmental processes (64).

General uncertainties suggest that further ethical analyses should also revolve around technical difficulties and susceptibility to abuse. These problems were already identified considering OMICS research, with the requirement for ethical and legal standards (65). Apparently, the issues of the reuse of identifiable material have become so difficult to handle in a research context that the cornerstone guideline for research ethics, namely the Declaration of Helsinki, is currently under revision (66). The proposed new text advocates for explicit advanced consent for any foreseeable reuse and it reiterates individuals’ right to alter their consent at any time or withdraw their material or data from datasets or biobanks. The ethics committee is supposed to monitor the ongoing use of the databases, in accordance with the current propositions of national institutions of ethics (64). At least two crucial questions remain open: (a) Which is a stronger patients empowerment tool: the informed consent for any data usage now and in the future or the anonymization of patients’ data? The two cannot go together for mere technical reasons. (b) Do the Ethics Committees have the capacity and expertise to perform this monitoring task, also demanded in the “systemic oversight” approach (60), in addition to all other responsibilities imposed on them?

Experts in our study recommended that national data ecosystems and public-private collaboration should be promoted in the EU. Higher representativity could be achieved through a new consciousness of data sharing and data altruism in organisations. The first steps are already made with the “Data Governments Act,” applicable since September 2023 that enables broader sharing and use of data, whilst GDPR focuses on a legal standardization to facilitate European research. However, it was pointed out that the regulation should keep evolving (67).

Broad consent for data use as part of the study design was appreciated to facilitate data usage and follow-up studies. The Medico-ethical literature on the issue is divided on this question (27). All interviewees agreed that aggregated anonymized data may be usable for additional projects depending on the purpose of the research without the need for a renewed informed consent procedure, provided that ethical principles are adhered to. This aligns with recent findings, as data donations for medical research were supported concerning patients’ perspectives (25). Further information on the patient and the use of his data is considered necessary and manageable through an app that provides security and privacy and gives the chance to actively deny or accept participation through push messages. This is discussed controversially in literature, as technical difficulties are emerging, not meeting requirements of privacy and alignment of the common good (68, 69). Legal experts implied special considerations. A retroactive redefinition of research purposes would be possible in their view if there is broad consent in place or complete anonymization can be assured; otherwise, renewed informed consent should be obtained, as the GDPR already demands the definition in advance (70). The general agreement could only be reached by addressing this question politically and through intended purposes if special public interests exist and the described requirements cannot be fulfilled, as it was not clear that full anonymization could be safely assured with OMICS research. This aligns with reflections, posed in literature, applying new methods and broader ethical principles for the new fields of using AI and managing data, as new questions arise for already existing material (71).

Small cohort size and confounding biases were identified as challenges; however, they were deemed manageable through a well-executed study design, the establishment of stringent protocols, and a precision medicine approach. Issues related to social justice were deemed either unsolvable or unnecessary to be addressed at the current stage. For SIRS in adults, differences in age, race, and sex were noted (72). Furthermore, the literature clearly shows the impacts of socioeconomic, ethnic, gender, and religious factors on clinical research (12). Some of our interview partners argue that participation biases are only one variable of many and should therefore be evaluated by researchers and encourage further studies. They should not be artificially separated as social justice issues from other biases, as altering in advance may have its own subjectivity. However, they could be addressed, by facilitating a broader collection of data in general, which aligns with ethical considerations of increasing accessibility in literature (73). The relation between socioeconomic status, cultural heterogeneity, diet, gut microbiome, and time dependency of OMICS status was exemplified, which must be addressed by medical professionals as they merge together and should not be constricted in advance through socioeconomic interpretations. Some of our interview partners shared a similar opinion that there should be no direct attempt to mitigate socioeconomic injustices through guidelines, politics, or legislation for studies, as it contradicts established ethical standards. According to the WMA Declaration on the rights of the patient, “every person is entitled without discrimination to appropriate medical care,” and when a choice between patients should be made, only medical criteria should be applied (74). In the same spirit, the European Charter of Patients’ Rights claims that “every individual has the right of access to the health services that his or her health needs require,” and that equal access without discrimination must be guaranteed. The same is valid for access to innovative procedures, including diagnostic ones (75). The limitations of the therapy approach through genetic diversity are not presented unanimously in the literature, though this challenge could be addressed by choosing smaller cohorts in the future and taking a further gender- or ethnicity-specific approach. The risk of biases, due to the heterogeneity of sampling, is not sufficiently addressed but could harm certain patient groups described (76).

All experts agreed that an interdisciplinary discourse and consensus should be promoted, addressing ethical, legal, and technical issues. This aligns with recommendations from studies rooted in computer science that address ethical issues of AI in medicine (58, 59). Apparently, the drive for new technologies in medicine needs the joint efforts and effective collaboration of many spheres of science as never before. Experts emphasised that OMICS research, AI application, and precision medicine are novel concepts, and therefore, guidelines are needed, but should be extremely broad-minded in their initial stages. It was emphasised that they should adapt and evolve alongside medicine, as it is a practical science and not solely focussed on the precautionary principle.

### 4.1 Limitations

There are several limitations to this qualitative research that need to be mentioned in order to properly assess its results. The first limitation is the low generalisation of the results. The study cohort was limited to a maximum of 10 participants. Thus, this does not allow for a generalisation from a global perspective. However, wide generalisation of the results was not the aim of our research. On the contrary, the goal was to present a subjective view from a group of stakeholders connected to the development and implementation of new diagnostic and therapeutic technology in healthcare practice and to analyse it from an ethical perspective. Furthermore, the size of the sample provides also an advantage—it allows a detailed inspection and assessment of individual views and opinions in a detailed way, which cannot be easily achieved through quantitative research methods. This approach is common for research in applied ethics and allows for a detailed inspection of individual views of interviewees on a particular topic as a first step towards an ethical assessment of a new technology. The limited number of interviews allows for a detailed inspection of interviewees’ perspectives and arguments, as well as the formulation and development of new topics for further research. A sample set of 10 interviews is not small for qualitative studies. Typically, most of the essential data in qualitative interviews can already be identified in a few initial interviews (77, 78). Data saturation achieved with the sample of 10 interviews was assessed within the team of researchers, who concluded that the gathered material provided essential information for the ethical analysis. Moreover, one of the limitations of the method of thematic analysis used in this research is the subjectivity of the coding system, which can be subject to individual interpretation. To avoid this issue, the content of the interviews was always analyzed and coded by at least two independent researchers. Differences in interpretation were discussed and resolved within the multidisciplinary team of researchers—which in itself can be a strength of the investigation. Furthermore, because the research questions do not specifically investigate the influence of individual characteristics of interviewees, i.e., age, gender, career path, or professional background, no such information was collected during the interviews.

An extension of the research with the use of other research methods could provide a valuable triangulation of our results and a wider generalization. However, such a correlation is not the purpose of a qualitative study and cannot be reached with the use of this method. Correlations between interviewees’ individual backgrounds and their presented opinions could be an interesting aim of further investigations, which, however, need to be conducted with the use of quantitative research methods.

## 5 Conclusion

We identified the process of consent, affecting the autonomy of children and legal guardians, as challenging for personalized SIRS research since new diagnostic procedures with data processing and AI must be included, increasing vastly the amount of information and the difficulty of understanding necessary to make an autonomous decision. Considering the well-known time limitations of emergency medicine, there is a clear need to establish guidelines for orientation. The importance of empowering the child was therefore pointed out. Instruments should be promoted to enhance understanding. As with every new technological development, ethical and legal challenges cannot be foreseen for SIRS personalized treatment. That being the case, experts emphasized the importance of extending the ethical-legal discourse beyond mere restrictions. The array of clinical scenarios, presented by SIRS, underscores the need for a broad scope of subjective interpretations provided by the physician. Accordingly, the organization of supervision should be reconsidered, and not limited only to the precautionary principle, which *per se* was seen as impeding both medical progress and clinical flexibility. Current guidelines lack an enabling approach. Thus, swift governmental reactions in this direction are essential. Measurements of cognitive abilities were seen to be negligible since they are not widely applicable and are not considered by legislation. The imminent health risks of OMICS research were deemed low, but issues of data protection and misuse were anticipated. Hence, new technologies were suggested to promote autonomy not only in a clinical context but also with respect to individual health data. The security of anonymization was questioned, and specific frameworks to prevent traceability should be established, particularly for research methodology. Social justice issues should not be artificially separated from other confounding biases, but rather be evaluated in the aftermath. To address these challenges, interpretation, monitoring and the establishment of guidelines should occur in an interdisciplinary discourse, whilst at the same time training of physicians in computer science and training of AI-developers in ethics should take place, both accompanied by a legal glimpse.

## Data Availability

The raw data supporting the conclusions of this article will be made available by the authors, without undue reservation.
